# Effect of radioprotective curtain length on the scattered dose rate distribution and endoscopist eye lens dose with an over-couch fluoroscopy system

**DOI:** 10.1007/s13246-024-01398-w

**Published:** 2024-03-14

**Authors:** Kosuke Matsubara, Asuka Nakajima, Ayaka Hirosawa, Ryo Yoshikawa, Nao Ichikawa, Kotaro Fukushima, Atsushi Fukuda

**Affiliations:** 1 Department of Quantum Medical Technology, Faculty of Health Sciences, Institute of Medical, Pharmaceutical and Health Sciences, Kanazawa University, 5-11-80 Kodatsuno, Kanazawa, Ishikawa 920-0942 Japan; 2 Department of Radiological Technology, School of Health Sciences, College of Medical, Pharmaceutical and Health Sciences, Kanazawa University, 5-11-80 Kodatsuno, Kanazawa, Ishikawa 920-0942 Japan; 3 Department of Medical Technology, Toyama Prefectural Central Hospital, 2-2-78 Nishinagae, Toyoma, Toyama 930-8550 Japan; 4 Department of Radiology, Kanazawa University Hospital, 13-1 Takaramachi, Kanazawa, Ishikawa 920-8641 Japan; 5 Department of Quantum Medical Technology, Division of Health Sciences, Graduate School of Medical Sciences, Kanazawa University, 5-11-80 Kodatsuno, Kanazawa, Ishikawa 920-0942 Japan; 6 Department of Radiological Technology, Faculty of Health Science, Kobe Tokiwa University, 2-6-2 Otani-Cho, Nagata-Ku, Kobe, Hyogo 653-0838 Japan; 7 Department of Radiological Sciences, School of Health Sciences, Fukushima Medical University, 10-6 Sakaemachi, Fukushima, Fukushima 960-1295 Japan; 8 Present Address: Division of Radiology, Shinshu University Hospital, 3-1-1 Asahi, Matsumoto, Nagano, 390-8621 Japan

**Keywords:** Radiation protection, Radiation measurement, Occupational exposure, Eye lens, Radioprotective curtain

## Abstract

Sufficient dose reduction may not be achieved if radioprotective curtains are folded. This study aimed to evaluate the scattered dose rate distribution and physician eye lens dose at different curtain lengths. Using an over-couch fluoroscopy system, dH*(10)/d*t* was measured using a survey meter 150 cm from the floor at 29 positions in the examination room when the curtain lengths were 0% (no curtain), 50%, 75%, and 100%. The absorbed dose rates in the air at the positions of endoscopist and assistant were calculated using a Monte Carlo simulation by varying the curtain length from 0 to 100%. The air kerma was measured by 10 min fluoroscopy using optically stimulated luminescence dosimeters at the eye surfaces of the endoscopist phantom and the outside and inside of the radioprotective goggles. At curtain lengths of 50%, 75%, and 100%, the ratios of dH*(10)/d*t* relative to 0% ranged from 80.8 to 104.1%, 10.5 to 61.0%, and 11.8 to 24.8%, respectively. In the simulation, the absorbed dose rates at the endoscopist’s and assistant’s positions changed rapidly between 55 and 75% and 65% and 80% of the curtain length, respectively. At the 0%, 50%, 75%, and 100% curtain lengths, the air kerma at the left eye surface of the endoscopist phantom was 237 ± 29, 271 ± 30, 37.7 ± 7.5, and 33.5 ± 6.1 μGy, respectively. Therefore, a curtain length of 75% or greater is required to achieve a sufficient eye lens dose reduction effect at the position of the endoscopist.

## Introduction

Ionizing radiation is used in medicine for diagnostic and therapeutic purposes and is now essential for the provision of quality medical care. To use radiation safely and effectively, it is important to ensure effective radiation protection for patients and healthcare workers. The number of fluoroscopic examinations of the biliary system and their contribution to the collective dose has decreased. However, the contribution of interventional radiology procedures performed by gastroenterologists to the collective dose has increased considerably [1].

The eye lens is sensitive to ionizing radiation, and radiation cataracts are visual disorders associated with exposure to ionizing radiation. Several studies have provided epidemiological evidence that lens opacification develops with lower radiation doses than previously thought [2–4]. The International Commission on Radiological Protection reduced the dose threshold for the development of cataracts from 2 to 0.5 Gy (with 90–95% confidence intervals, including zero dose). It is recommended that the occupational equivalent dose limit for the eye lens should be revised to 20 mSv/y, averaged over a five-year period, with no single year exceeding 50 mSv [5]. A previous study showed that the prevalence of radiation-related posterior lens opacification was higher among interventional cardiologists and nurses than among controls [6]. Furthermore, the existence of a dose threshold for cataracts remains unclear, and no threshold for cataracts may be biologically plausible [7, 8]. Therefore, it is important for medical staff to protect their eye lenses from radiation cataracts by minimizing the risk of stochastic damage to the eye lens from ionizing radiation and to maintain their eye lens doses below the threshold.

During fluoroscopy-guided endoscopic procedures such as endoscopic retrograde cholangiopancreatography (ERCP), not only gastroenterologists but also other medical staff members, such as nurses and radiological technologists, have the potential to receive high radiation doses to their eye lenses because they must stand close to the patient during the procedure [9]. In general, the endoscopist stands closest to the patient, leading to the highest radiation exposure. If the patient is unstably sedated and there is a lot of body movement, the nurse should stand closest to the patient for restraint [10]. The workspace of an over-couch fluoroscopy system is larger than that of an under-couch one, but more backscattered radiation reaches to the upper bodies of the gastroenterologists and other medical staff members, resulting in a higher radiation dose to the eye lenses than an under-couch one [10, 11]. The use of radioprotective curtains in over-couch fluoroscopy systems is highly effective in reducing radiation exposure to physicians and other staff members [12–14]. They consist of hood and four radioprotective sheets, and the hood is placed over the X-ray tube, while the radioprotective sheets are hang down to the surface of the system couch [15]. Minami et al. [12] showed that the radiation dose to the endoscopist and other staff members during one ERCP procedure decreased by 87.5 and 68–85%, respectively, when radioprotective curtains were used. Yamada et al. [16] showed that the scattered radiation dose at the medical staff location decreased by 86.4–91.2% at the height of 150 cm. On the other hand, radioprotective curtains can be folded in the middle, and a case of their inappropriate use has been reported [17]. Another study found that folding the curtains at 50% of its length reduced scattered radiation by only ≤ 10% at the position of the endoscopist [13]. However, the relationship between curtain length and dose reduction rate by position has not been evaluated in detail. Understanding this relationship is necessary so that radiation protection curtains can be used effectively in clinical practice.

Therefore, this study aimed to evaluate the scattered dose rate distribution and endoscopist eye lens doses at different radioprotective curtain lengths.

## Materials and methods

### Measurement of dH*(10)/d*t* by survey meter

**Fig. 1:**
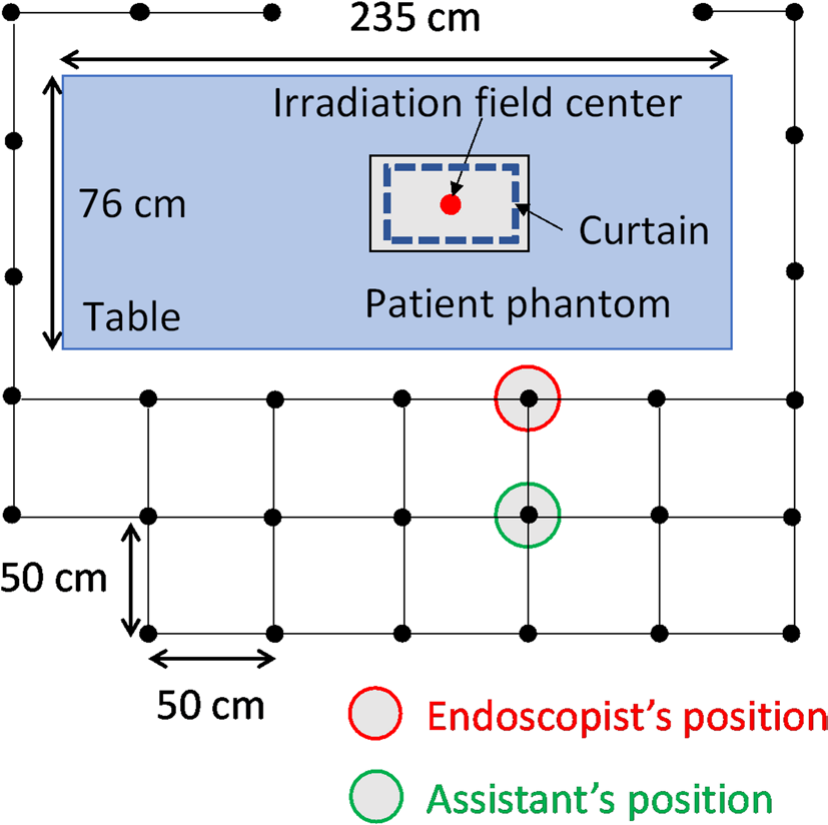
Measurement positions of dH*(10)/d*t* in the examination room. The red and green circles show the positions where the endoscopist and assistant generally stand during endoscopic retrograde cholangiopancreatography

As the gold-standard method for evaluating scattered dose, an over-couch fluoroscopy system (SONIALVISION G4; Shimadzu, Kyoto, Japan) and an anthropomorphic body phantom (Alderson Phantom Patient; The Phantom Laboratory, Salem, NY, USA) was used to measure the ambient dose equivalent rate at 10 mm depth (dH*(10)/d*t*) as a scattered dose rate using a survey meter (ICS-1323; Hitachi, Tokyo, Japan) at 29 positions 150 cm from the floor (assuming the position of the eye lenses), as shown in Fig. 1, to understand the scattered dose distribution in the examination room in detail, which is helpful for estimating the eye lens doses of the endoscopists, assistants, and other staff members depending on their standing positions. The survey meter had been calibrated against Cs-137 γ-rays (662 keV) and was directed toward the patient phantom when measuring dH*(10)/d*t* at each position.

**Fig. 2:**
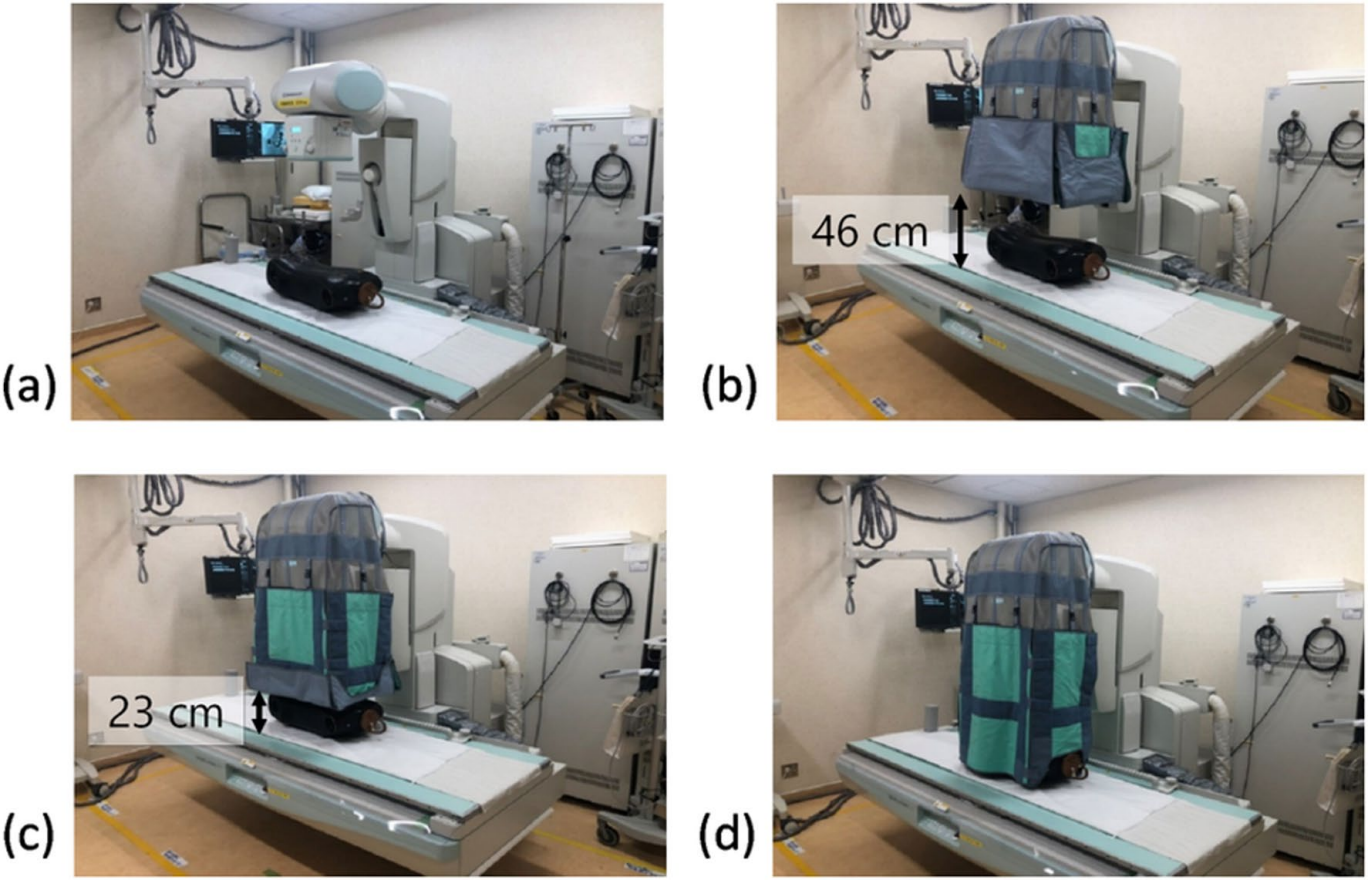
Four different curtain lengths for the measurement of dH*(10)/d*t*: **a** 0% (no curtain), **b** 50%, **c** 75%, **d** 100%. When it was 50%, the curtain edge was 46 cm above the table. When it was 75%, the curtain edge was located 23 cm above the table

A radioprotective curtain with 0.125 mmPb (TI-13; MAEDA & CO., LTD, Tokyo, Japan), which could be folded at any height using Velcro, was placed over the X-ray tube housing. The measurements of dH*(10)/d*t* were repeated by changing the length of the curtain to 0% (no curtain), 50%, 75%, and 100%, as shown in Fig. 2. The following fluoroscopy conditions, which are commonly used for ERCP at our facility, were used: a tube voltage of 96–99 kV and tube current of 2.6 mA (with automatic brightness control), pulse rate of 7.5 pulses per second (pps), field size of 9 inches (22.9 × 22.9 cm), source-to-image receptor distance (SID) of 120 cm, and a table height of 75 cm from the floor. The variation in tube voltage (96–99 kV) was not due to the presence or absence of radiation protection curtains but to changes over time.

Dose distributions were drawn using a contour plot in OriginPro 2022 (OriginLab, Northampton, MA, USA), which uses Delaunay triangulation to connect the nearest data points to form a triangle. The dose reduction rate (DR [%]) of the curtain was calculated using the following equation:

1 $${\text{DR}}\left[\%\right]=\frac{{{{\text{d}}H}^{*}\left(10\right)}_{0}/{\text{d}}t-{{{\text{d}}H}^{*}\left(10\right)}_{x}/{\text{d}}t}{{{{\text{d}}H}^{*}(10)}_{0}/{\text{d}}t}\times 100,$$

where dH*(10)_0_/d*t* and dH*(10)_*x*_/d*t* represent dH*(10)/d*t* at 0% and *x*% curtain lengths, respectively.

### Calculation of the absorbed dose by Monte Carlo simulation

When measuring dH*(10)/d*t* using a survey meter, the measurer is constantly exposed to scattered radiation. In addition, it can be challenging to fold radioprotective curtains to a certain length due to their thickness. Therefore, to verify the measured dH*(10)/d*t* by the survey meter and to obtain the detailed scattered doses at the positions of the endoscopist and assistant, the absorbed dose in the air at these positions was calculated using a Monte Carlo simulation code (PHITS version 3.29 [18]; Japan Atomic Energy Agency, Tokai, Japan) by changing the curtain length from 0 to 100% in 5% increments. The simulated radioprotective curtain was made of 0.125 mm lead. The curtains placed in the patient’s longitudinal direction were 92 cm long and 50 cm wide, and those placed in the patient’s lateral direction were 72 cm long and 40 cm wide (the lateral sides were 20 cm shorter at the bottom than the front and back sides). The curtains were attached to a model X-ray tube housing when the curtain length was ≥ 5%.

As an alternative to an anthropomorphic body phantom, an elliptical phantom with a height of 20 cm, width of 30 cm, and length of 60 cm filled with International Commission on Radiation Units and Measurements (ICRU) soft tissue (tissue equivalent material with a density of 1.00 g/cm^3^ and a mass composition of 76.2% oxygen, 11.1% carbon, 10.1% hydrogen, and 2.6% nitrogen) [19] was used. The phantom was positioned on a model table immediately below the X-ray source in the X-ray tube housing. The outside and interior of the table consisted of carbon fiber (density of 1.7 g/cm^3^) and Styrofoam (density of 0.015 g/cm^3^), respectively. As in the measurement of dH*(10)/d*t*, the SID and the table height were set to 120 and 75 cm, respectively, and the field size was set to 9 inches (22.9 × 22.9 cm).

Two 1 cm^3^ air-filled regions were set 150 cm above the floor at the positions of the endoscopist and assistant to calculate the absorbed doses in the air [Gy/source]. A “T-Deposit” tally, which calculates the energy deposited to the specified region, was used for the absorbed dose calculation. The number of photons for each calculation was between 6.5216 × 10^9^ and 5.17226 × 10^10^ after repeating calculations with a relative standard error of ≤ 1%. The photon cut-off energy was set to 1 keV. The DR [%] of the curtains was calculated using the following equation:

2 $${\text{DR}}\left[\%\right]=\frac{{D}_{0}-{D}_{x}}{{D}_{0}}\times 100,$$

where *D*_0_ and *D*_*x*_ represent the absorbed doses at 0 and *x*% curtain lengths, respectively.

X-ray-Spectrum-2 [20] was used to calculate the X-ray energy spectra. This software generates X-ray energy spectra from a tungsten target based on the generator type, peak tube voltage, and total filtration using Tucker’s formula [21] or Birch and Marshall’s formula [22]. In the present study, the X-ray energy spectrum was calculated based on Tucker’s formula for a tungsten target at a tube voltage of 98 kV, target angle of 12°, and total filtration of 3.5 mmAl.

### Measurement of the eye lens dose for the endoscopist phantom

**Fig. 3:**
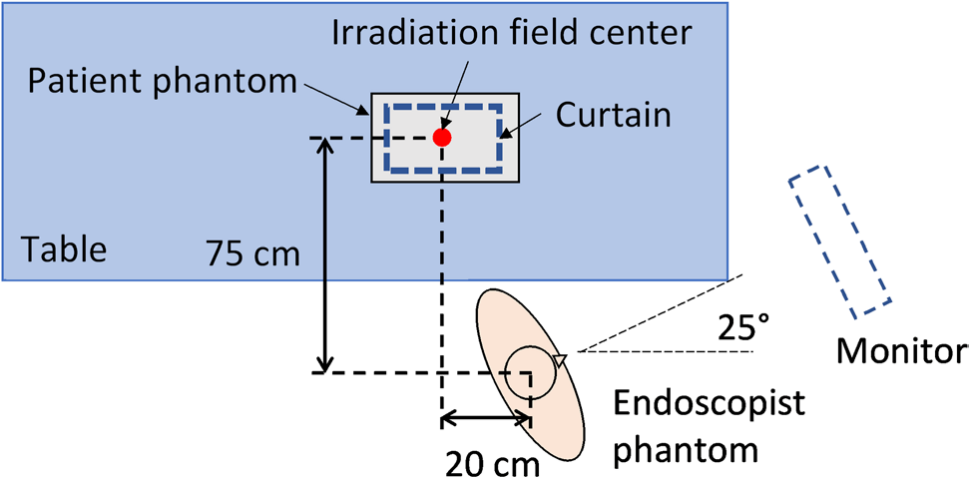
Arrangement of the patient and endoscopist phantoms. The endoscopist phantom was placed 75 cm behind and 20 cm to the right of the irradiation field center at an angle of 25° from the horizontal line

An endoscopist phantom and small optically stimulated luminescence (OSL) dosimeters (nanoDot; Landauer, Glenwood, IL, USA) were used to estimate eye lens doses for the phantom, as well as to validate the dH*(10)/d*t* measured by the survey meter and to verify the effectiveness of combining radioprotective curtains and goggles. The endoscopist phantom, which consisted of a head phantom (ACS-A; Kyoto Kagaku, Kyoto, Japan) and a custom-made chest phantom, was placed on a height-adjustable table (ERD-GAP1LMN; Sanwa Supply, Okayama, Japan) 75 cm behind and 20 cm to the right of the irradiation field center at an angle of 25° from the horizontal line, as shown in Fig. 3. The height of the eye lenses was adjusted to 150 cm above the floor using a height-adjustable table. Fluoroscopy was performed for 10 min on the anthropomorphic body phantom (Alderson Phantom Patient; The Phantom Laboratory) under the same fluoroscopic conditions as the dH*(10)/d*t* measurements.

**Fig. 4:**
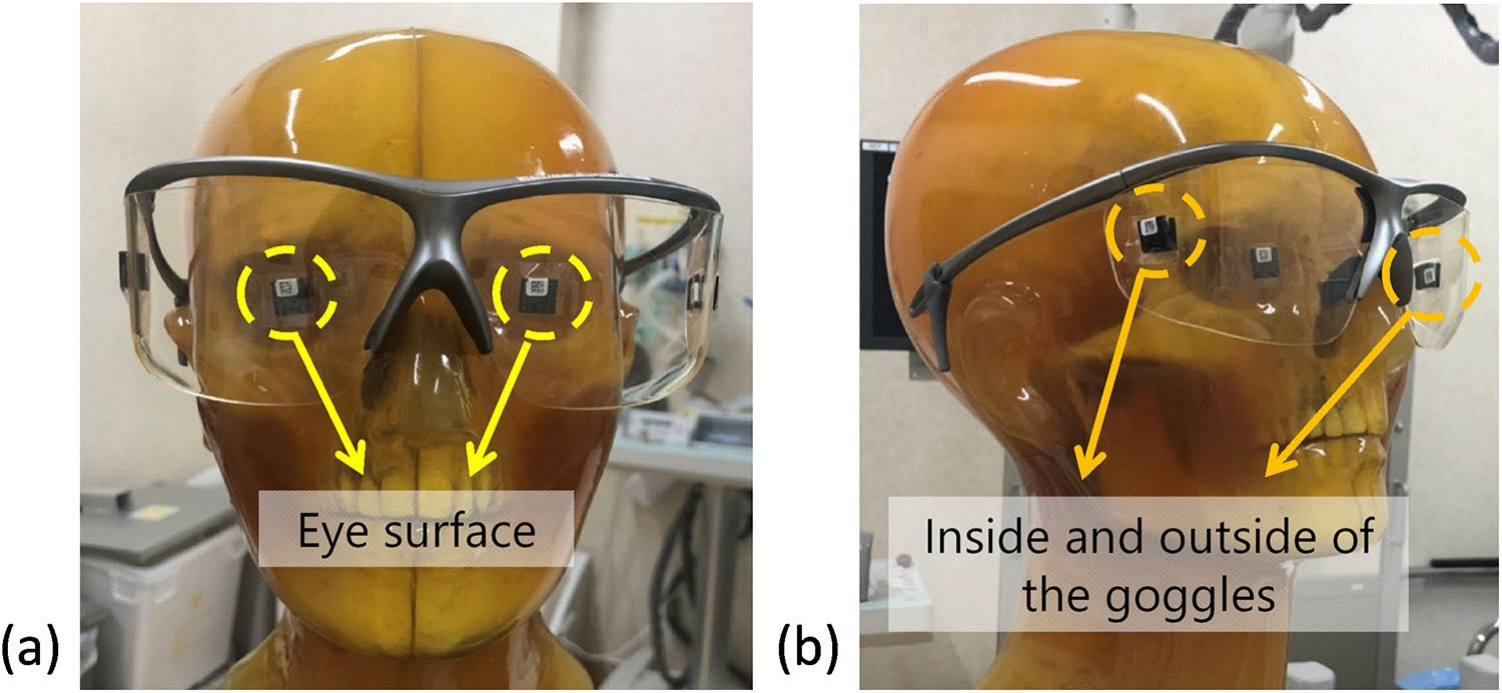
Positions of the small dosimeters for the measurement of the eye lens dose for the endoscopist phantom. They were attached to the surface of the eyes on the left and right sides and both the inside and outside of the radioprotective goggles with 0.07 mmPb on the left and right sides. The dosimeters on the inside and outside of the radioprotective goggles were superimposed through the goggles: **a** frontal view and **b** lateral view

Prior to use, the small OSL dosimeters were initialized by exposure to visible light emitted from a mammography illuminator (ORS-M1E B4; Orion Electric, Nagoya, Japan) for over 6 h, and the initial air kerma was obtained by averaging three readings using a reader (microSTAR; Landauer) calibrated against 80 kV X-rays. The small OSL dosimeters were attached to the surface of the eyes on both sides and on both the inside and outside of the radioprotective goggles made of leaded acrylic with 0.07 mmPb (HF-400S; Toray Medical, Tokyo, Japan), as shown in Fig. 4. The air kerma measurement was repeated three times under the same curtain length using separate sets of small OSL dosimeters for the four different curtain lengths (0%, 50%, 75%, and 100%). After irradiation, the air kerma was obtained by averaging three readings using a reader. The net air kerma was obtained by subtracting the initial air kerma from the air kerma after irradiation. The DR [%] of the curtains was calculated using the following equation:

3 $${\text{DR}}\left[\%\right]=\frac{{K}_{0}-{K}_{x}}{{K}_{0}}\times 100,$$

where *K*_0_ and *K*_*x*_ represent the net air kerma at 0 and *x*% curtain lengths, respectively.

Statistical analysis of the air kerma at different curtain lengths was performed using a one-way analysis of variance, followed by Tukey’s multiple comparison test, using SPSS Statistics version 29 (IBM, Chicago, IL, USA). The significance level was set to 5%.

## Results

### dH*(10)/d*t* distribution by the survey meter

**Fig. 5:**
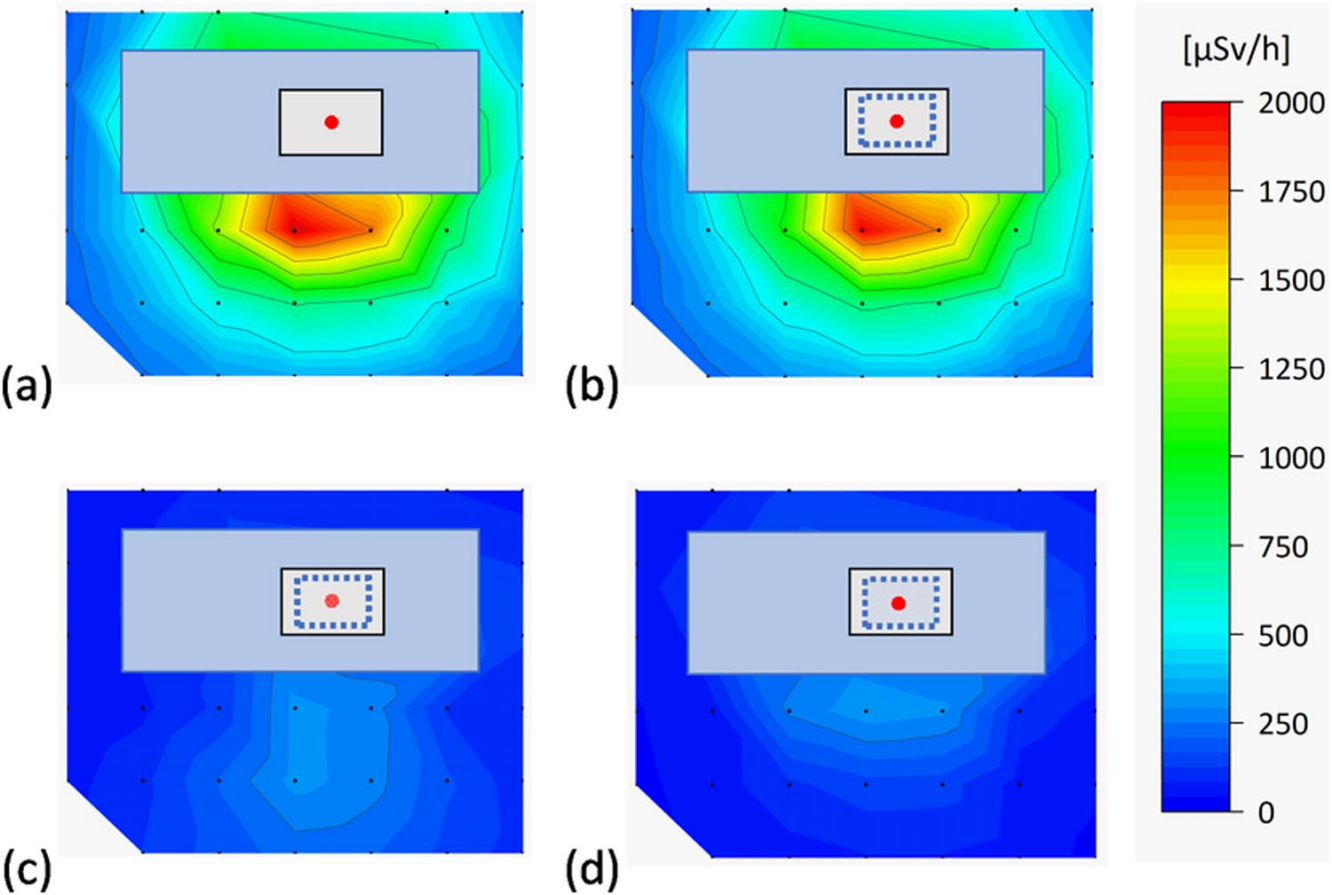
The dH*(10)/d*t* distribution maps for the different curtain lengths: **a** 0% (no curtain), **b** 50%, **c** 75%, **d** 100%

The dH*(10)/d*t* distribution results by curtain length are shown in Fig. 5. At curtain lengths of 50%, 75%, and 100%, the ratios of dH*(10)/d*t* relative to 0% at all measurement points ranged from 80.0 to 104.1%, 10.5 to 61.0%, and 11.8 to 24.8%, respectively.

At a curtain length of 50%, the dose rate was the highest near the X-ray tube and decreased with increasing distance. However, at a curtain length of 75%, the dose rate tended to be high even at positions away from the X-ray tube, including the position of the assistant.

### dH*(10)/d*t* at certain positions by the survey meter

The dH*(10)/d*t* for the different curtain lengths and DR [%] relative to the 0% curtain length at the positions of the endoscopist and assistant and the position of the maximum dose rate are shown in Table 1. At a curtain length of 50%, the dH*(10)/d*t* at the position of the endoscopist and the position of the maximum dose rate were slightly lower than 0% (1742 μSv/h against 1772 and 1990 μSv/h against 2039 μSv/h, respectively). However, the dH*(10)/d*t* at the position of the assistant was slightly higher than 0% (721 μSv/h against 696 μSv/h). The dH*(10)/d*t* at the positions of the endoscopist and assistant and the position of the maximum dose rate decreased sharply between the 50 and 75% curtain lengths (from 1742 to 274 μSv/h, 721 to 270 μSv/h, and from 1990 to 308 μSv/h, respectively), and the DR [%] relative to 0% at these positions increased sharply (from 1.69 to 84.5%, from − 3.59 to 61.2%, and from 2.40 to 84.9%, respectively).

**Table 1:** dH*(10)/d*t* for different curtain lengths and the dose reduction rate relative to the 0% curtain length at the positions of the endoscopist and assistant and the position of the maximum dose rate

Curtain length	dH*(10)/d*t* [μSv/h]			Dose reduction rate [%]		
	Endoscopist	Assistant	Maximum	Endoscopist	Assistant	Maximum
0%	1772	696	2039			
50%	1742	721	1990	1.69	− 3.59	2.40
75%	274	270	308	84.5	61.2	84.9
100%	300	145	320	83.1	79.2	84.3

### Absorbed doses at certain positions by simulation

**Fig. 6:**
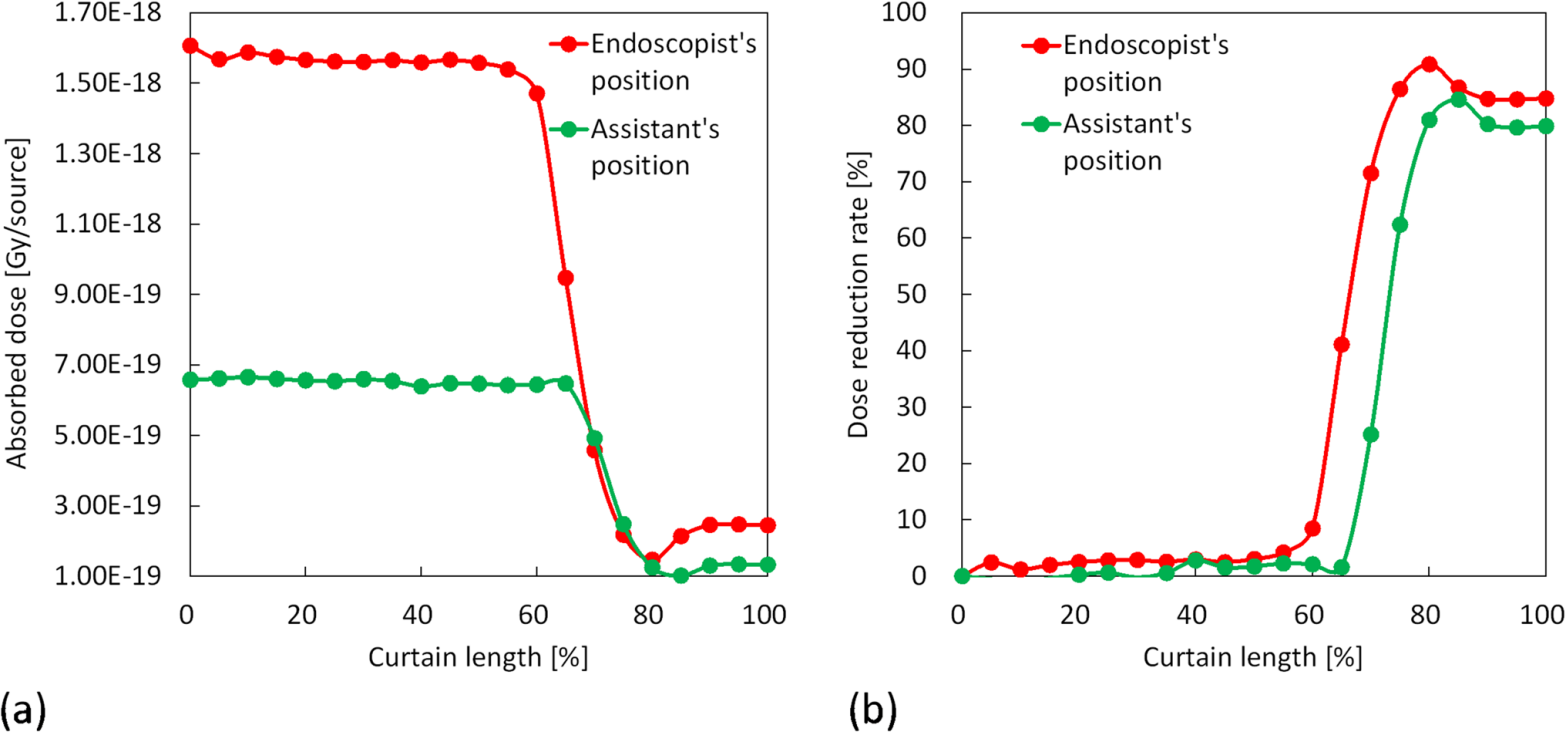
The absorbed doses for the different curtain lengths and dose reduction rates relative to the 0% curtain length at the positions of the endoscopist and assistant obtained by simulation: **a** absorbed doses, **b** dose reduction rates

The absorbed doses in the air and the DR [%] relative to the 0% curtain length for the different curtain lengths at the positions of the endoscopist and assistant obtained by the simulation are shown in Fig. 6. The DR [%] increased sharply between 55 and 75% of the curtain length at the position of the endoscopist (from 4.2 to 86.5%) and between 65 and 80% at the position of the assistant (from 1.6 to 81.0%). Moreover, the DR [%] became almost constant at longer curtain lengths. The highest DRs [%] were observed at 80% and 85% in the positions of the endoscopist and assistant, respectively.

### Eye lens dose for the endoscopist phantom

**Fig. 7:**
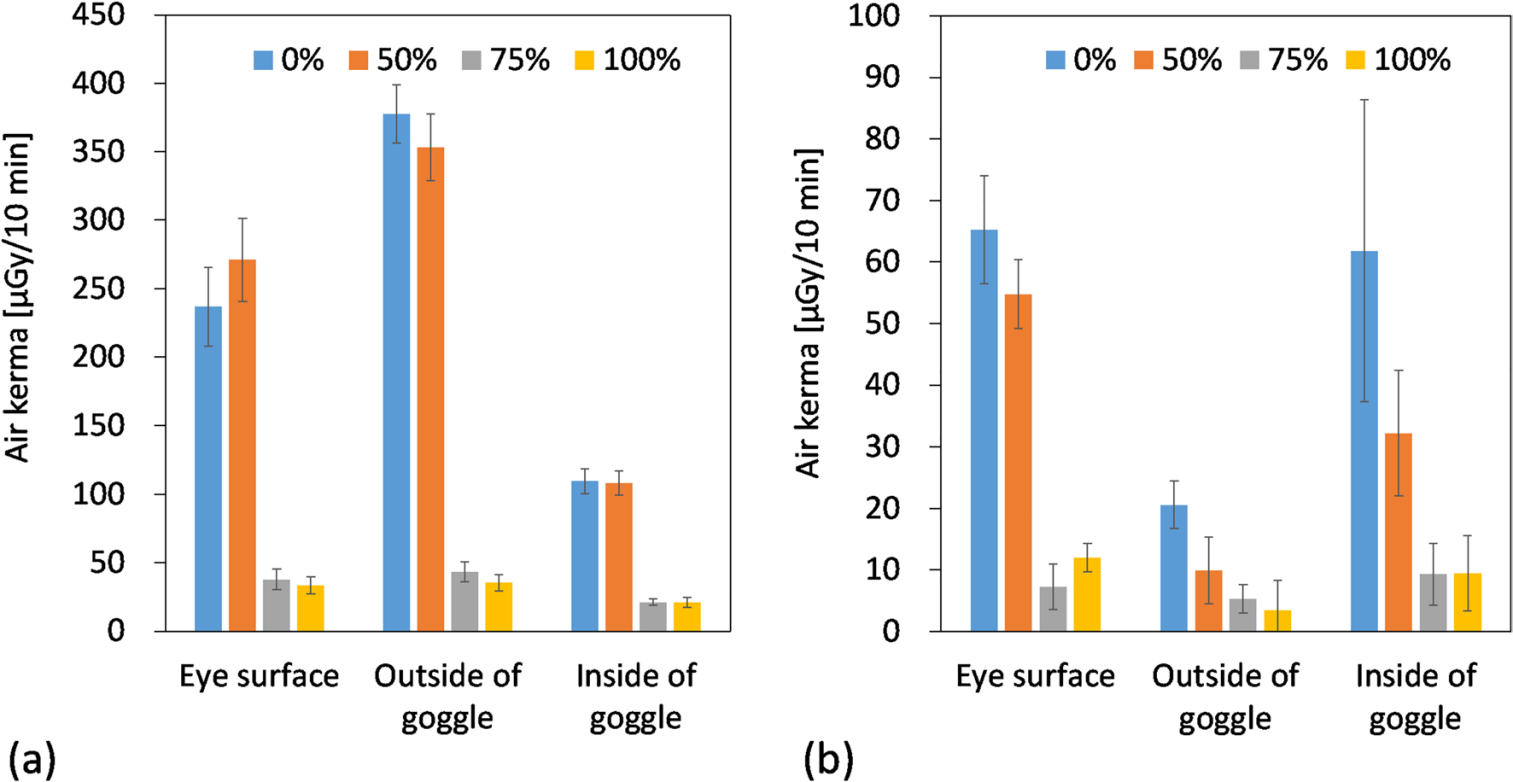
The eye lens doses for the endoscopist phantom for the different curtain lengths: **a** left side, **b** right side

The eye lens doses for the endoscopist phantom, which are expressed by the air kerma, are shown in Fig. 7. On the left side, which was near the X-ray tube, the air kerma at all the measurement points decreased significantly between 50 and 75% of the curtain length (*p* < 0.001). On the right side, which was far from the X-ray tube, the air kerma measured on the eye surface also decreased significantly between 50 and 75% of the curtain length (*p* < 0.01). At all the curtain lengths, the air kerma measured outside the radioprotective goggles was significantly lower than that measured inside the radioprotective goggles (*p* < 0.05).

## Discussion

Radioprotective curtains have been found to reduce physician eye lens doses in over-couch X-ray tube geometry; however, a sufficient dose reduction effect may not be obtained if the curtains are folded. Therefore, this study evaluated the scattered dose rate distribution and endoscopist eye lens doses at different radioprotective curtain lengths to clarify the relationship between radioprotective curtain length and DR [%] by position. At curtain lengths of 50%, 75%, and 100%, the ratios of dH*(10)/d*t* relative to 0% at all measurement points ranged from 80.0 to 104.1%, 10.5 to 61.0%, and 11.8 to 24.8%, respectively. The dH*(10)/d*t* at the positions of the endoscopist and assistant and the position of the maximum dose rate decreased sharply between the 50 and 75% curtain lengths (from 1742 to 274 μSv/h, 721 to 270 μSv/h, and from 1990 to 308 μSv/h, respectively). In the simulation, the DR [%] relative to the 0% curtain length at the positions of the endoscopist and assistant increased rapidly between 55 and 75% (from 4.2 to 86.5%) and 65 and 80% (from 1.6 to 81.0%) of the curtain lengths, respectively, was the highest at 80% (90.9%) and 85% (84.6%) of the curtain lengths, respectively, and became almost constant at longer curtain lengths. The highest DR [%] was observed at 80 and 85% at the positions of the endoscopist and the assistant, respectively, because folding curtain partially doubled the shielding effect. The air kerma at the left eye surface of the endoscopist phantom during 10-min fluoroscopy decreased significantly between 50 and 75% of the curtain length (from 271 ± 30 to 37.7 ± 7.5 μGy, *p* < 0.001).

Morishima et al. [13] stated that the curtains should always be unfolded to provide greater protection. However, this study revealed that the curtain length where the DR [%] changed rapidly differed according to position and that curtain lengths of 75 and 80% were required at the positions of the endoscopist and assistant, respectively, to achieve a sufficient eye lens dose reduction effect. This indicates that even if the curtain is folded to some extent, a sufficient dose reduction effect can be obtained at the positions of the endoscopist and assistant. In other words, a sufficient reduction in the eye lens dose depends on the presence of a curtain between the eye lenses and the patient, which is a major source of scattered radiation. The present study showed that a 50% curtain length reduced the scattered dose rate by only 20.0% at most, indicating that this length did not significantly affect the dose reduction for the eye lenses of physicians and other staff members in the examination room. It was also revealed that the radioprotective curtain did not significantly reduce the scattered radiation from the X-ray tube because a mesh material was used for the part that covered the X-ray tube housing for weight reduction and heat dissipation. A study by Nakagami et al. [23] showed a change in the scattered dose distribution when a custom-made lead radioprotective curtain with a 30 cm length was used; however, they measured the scattered dose distribution only near the head of the physician. We believe that their results differed from the current study as we measured the scattered dose distribution 150 cm above the floor.

Several types of radioprotective shields have been installed in examination rooms for use in conjunction with an over-couch X-ray tube geometry. For example, a prospective study showed that a custom-made 2 mmPb mobile shield with a 0.5 mmPb window, which was placed between the endoscopist and the patient, reduced the radiation dose by approximately 1/40 (DR [%] of 97.5%) during ERCP [24]. Radioprotective shields have an advantage in that the DR [%] is high if they are appropriately placed between the endoscopist and the patient. The disadvantages of radioprotective shields are that they sometimes interfere with the procedures, and physicians and other staff members cannot respond quickly if the patient’s condition changes or the patient moves suddenly. In addition to the radioprotective shields, reducing the pulse rate during fluoroscopy is also effective in reducing eye lens doses for physicians and other staff members. Nagamoto et al. [17] showed that the median d*H*_p_(3)/d*t* on the inner side of the lead glasses for the gastroenterologist during ERCP procedures reduced from 3.7 to 1.4 μSv/min after explaining the proper use of the radioprotective curtains to them and urging them to reduce the pulse rate from 15 to 7.5 pps. It is also important to develop a strategy before inserting the scope as the extension of the procedure time leads to an extension of the fluoroscopy time and an increase of the radiation exposure [25].

From the air kerma results measured on the left outside and inside of the radioprotective goggles worn by the endoscopist phantom with the 0% curtain length, the dose reduction effect of the 0.07 mmPb radioprotective goggles was estimated to be 71.0%. However, the DR [%] for the eye lens with the radioprotective goggles is expected to be less than 71.0% because of factors such as scattered X-rays entering through gaps in the radioprotective goggles. On the other hand, the dH*(10)/d*t* at the 100% curtain length was 83.1 and 79.2% lower than at the 0% curtain length at the positions of the endoscopist and assistant, respectively. A study by Imai et al. [26] showed higher dose reduction effects to the eye lenses for radioprotective goggles/glasses and radioprotective curtains than in this study: up to 80.0 and 96.8%, respectively. The radioprotective goggles used in this study were of the over-glass type, and there may have been larger gaps between the phantom and the goggles in this study. In their study, 0.25 mmPb radioprotective curtains were used, which was higher than this study (0.125 mmPb). Although there are differences in dose reduction rates in both studies, these findings indicate that the radioprotective curtains can provide a higher dose reduction effect than the radioprotective goggles to the eye lenses because the radioprotective curtains can reduce the number of scattered X-rays at the positions of the endoscopist and assistant directly.

The estimated annual *H*_p_(3) for gastroenterologists who perform fluoroscopy-guided endoscopic procedures using an over-couch fluoroscopy system with 1 h fluoroscopy per month based on the results of *H*_p_(3) measured on the eye surfaces of the endoscopist phantom is shown in Table 2. Here, 1.28 Sv/Gy, which is for a narrow spectrum series of N-40 and an incident angle of 0º, was used as *h*_p*K*_(3) to convert the air kerma to *H*_p_(3) for the cylinder phantom consisting of ICRU tissue [27]. Even with radioprotective goggles, it is estimated that *H*_p_(3) may exceed 20 and 3 mSv/yr when the curtain length is 0% or 50% and 75% or 100%, respectively. Therefore, folding radioprotective curtains too much impairs their protective effects. Even if the length of the radioprotective curtains is 75% or greater, it is recommended for endoscopists to wear radioprotective goggles in conjunction with using radioprotective curtains during fluoroscopy-guided endoscopic procedures to further reduce their eye lens doses because 3 mSv/yr is still higher than many other radiation workers. The same goes for nurses who often have the opportunity to restrain patients during fluoroscopy-guided endoscopic procedures because they should stand closest to the patient many times. According to the meta-analysis by Menon et al. [28], ocular radiation exposures can be high in gastroenterologists who perform > 200 ERCP procedures/yr and those who are routinely involved in complex ERCP or interventional endoscopy involving prolonged fluoroscopy. Therefore, we especially recommend them to wear radioprotective goggles in conjunction with using radioprotective curtains. Garg et al. [29] performed single-center, prospective observational study by utilizing C-arm fluoroscopy system with the under-couch X-ray tube geometry, and the radiation dose rate to the eye lens during ERCP was shown to be 0.34 mSv/h for attending endoscopists. From the estimated annual *H*_p_(3) for gastroenterologists shown in Table 2, the *H*_p_(3) per hour for the left eye surface of the operator phantom was estimated to be 1.81, 2.08, 0.29, and 0.26 mSv/h at the curtain lengths of 0%, 50%, 75%, and 100%, respectively. Although the current study only included experiments with phantoms, it is suggested that the combination of radioprotective goggles and radioprotective curtains with a curtain length of 75% or greater with an over-couch X-ray tube geometry will result in an endoscopist’s eye lens dose approximately equivalent to that when using an under-couch X-ray system. Hayashi et al. [30] performed nationwide multi-center observational study to prospectively collect radiation doses for fluoroscopy-guided gastrointestinal procedures in Japan, and they showed that 65% of fluoroscopic systems participating in their study were used by the over-couch X-ray tube geometry. However, the ideal situation is to use by the under-couch X-ray tube geometry rather than the over-couch one to reduce eye lens doses to medical staff members. If the over-couch X-ray tube geometry is unavoidable, it is essential to use radioprotective curtains with a curtain length of 75% or greater. Ikezawa et al. [31] performed multi-center, prospective observational study, and the median estimated annual radiation doses to the eye lens during ERCP was shown to be 3.7 mSv for operators, which was approximately equivalent to the *H*_p_(3) for the left eye surface of the operator phantom at the curtain lengths of 75% and 100% in the present study (3.47 and 3.08 mSv, respectively).

**Table 2:** Estimated annual *H*_p_(3) for gastroenterologists who perform fluoroscopy-guided endoscopic procedures using an over-couch fluoroscopy system with 1 h fluoroscopy per month

Curtain length	*H*_p_(3) [mSv/y]	
	Right eye surface	Left eye surface
0%	6.02	21.8
50%	5.05	25.0
75%	0.67	3.47
100%	1.11	3.08

This study has several limitations. First, because it included only phantom-based measurements and simulation data, the findings need to be validated in a prospective clinical study. Second, the present study used specific conditions and devices, including the fluoroscopic conditions, over-couch fluoroscopy system, and radioprotective curtains, which could affect the results. Third, the standing positions of the endoscopist and assistant were considered in only one position. Although the standing positions were generally constant, the required curtain lengths would have been different if the standing positions were different. However, the present study also measured dH*(10)/d*t* distribution in the examination room in detail, which is helpful for estimating the eye lens doses of the endoscopists, assistants, and other staff members depending on their standing positions. Finally, all measurements and simulations were performed 150 cm above the floor. The eye lens doses for the endoscopist phantom were obtained by adjusting the height of the eye lens to 150 cm above the floor. If the heights of the eye lenses of the endoscopist and assistant are less than 150 cm, curtain lengths of 75 and 80%, respectively, may not be sufficient to provide an adequate dose reduction. Conversely, if the heights of the eye lenses of the endoscopist and assistant are greater than 150 cm, curtain lengths of less than 75 and 80%, respectively, may provide an adequate dose reduction.

## Conclusion

This study evaluated the scattered dose rate distribution and endoscopist eye lens doses at different radioprotective curtain lengths. The curtain length and DR [%] varied depending on the position. If the lens height of the endoscopist is 150 cm from the floor, the curtain length should be 75% or greater at the position of the endoscopist to achieve sufficient eye lens dose reduction. Even if the length of the radioprotective curtains is 75% or greater, it is recommended for endoscopists to wear radioprotective goggles in conjunction with using radioprotective curtains during fluoroscopy-guided endoscopic procedures to further reduce their eye lens doses.

## Data Availability

The data cannot be made publicly available upon publication because no suitable repository exists to host them. The data supporting the findings of this study are available from the authors upon reasonable request.
